# A new *Acartophthalmites* Hennig from Eocene Baltic amber (Diptera, Acalyptratae)

**DOI:** 10.3897/zookeys.737.20639

**Published:** 2018-02-13

**Authors:** Ricardo Pérez-de la Fuente, Christel Hoffeins, Jindřich Roháček

**Affiliations:** 1 Oxford University Museum of Natural History, Parks Road, Oxford, OX1 3PW, UK; 2 Liseistieg 10, D-22149 Hamburg, Germany; 3 Department of Entomology, Silesian Museum, Nádražní okruh 31, CZ-746 01 Opava, Czech Republic

**Keywords:** fossil, new species, Opomyzoidea, taxonomy, true flies

## Abstract

A new fossil fly species, *Acartophthalmites willii* **sp. n.** (Diptera: Acalyptratae: Opomyzoidea) from Baltic amber (Eocene, 56−33.9 Ma), is described based on a male originally assigned by Hennig (1969) to *A. tertiaria* Hennig, 1965, who erroneously also referred to it in the same work as “*A. electrica* Hennig” (unavailable name). The new species, representing the third named species of the extinct genus with unclear familial relationships *Acartophthalmites* Hennig, 1965, is herein described and illustrated in detail, and its systematic implications and relationships are discussed. From the morphological standpoint, the new species represents an intermediate form between the two formerly described species within the genus, therefore expanding the character combination diversity in this lineage of acalyptrate flies. The genus *Acartophthalmites* is considered to be most closely related to Clusiidae and, therefore, it is herein tentatively classified within the superfamily Opomyzoidea. The current work takes part of an effort to review the *Acartophthalmites* diversity in order to gain knowledge on the morphological data from the specimens described within the genus and ultimately enable a reliable analysis of its phylogenetic relationships with other acalyptrates.

## Introduction

The Eocene Baltic amber is the World’s richest source of fossil acalyptrate Diptera (see von Tschirnhaus and Hoffeins 2009). Even so, the diversity of this group of flies in Baltic amber has been insufficiently studied for the majority of its families. The essential works on acalyptrates in Baltic amber by Hennig (1965, 1966, 1967, 1969, 1971, 1972) proved to be only the first attempts to understand the communities living in the mid Eocene amber forests and adjacent habitats. For instance, the recent revision of Anthomyzidae (Roháček 2013, 2014) showed that the diversity of this family in the Baltic amber forest could have been even greater than it is across the whole Europe today.

Although the acalyptrate fossil genus *Acartophthalmites* Hennig, 1965 was established as monotypic (for *A. tertiaria* Hennig, 1965 from Baltic amber), recent revisionary efforts have revealed that this lineage of unclear familial affiliation could be richer in species than anticipated. Indeed, Roháček (2016), when describing the second species known of the genus, *A. clusioides* Roháček, 2016, noted that Hennig (1965, 1969) probably included several different species within *A. tertiaria*. Unfortunately, most of the specimens designated by Hennig as paratypes of *A. tertiaria* (all females) are no longer available for study and therefore their true identity cannot be confirmed.

A specimen in the Haren Collection from the Museum of Comparative Zoology, Harvard University (MCZ) on which Hennig (1969, p. 18) based his subsequent description of the male of *A. tertiaria* is the subject of the present contribution. The detailed examination of this relatively well-preserved amber inclusion confirmed the assumption made by Roháček’s (2016: p. 418) that this specimen was not conspecific with the *A. tertiaria* holotype. As the male in question is also distinct from *A. clusioides*, it represents a new species and the third *Acartophthalmites* species to date described. The new species is compared with the two other known species classified within the genus and its affinities are discussed.

## Materials and methods

The specimen was examined using a Leica MZ16 stereomicroscope and an Olympus BH-2 compound microscope. Pictures were taken with a Canon 6D camera attached to the stereomicroscope. Pencil drawings were made with the aid of the camera lucida attached to both the stereomicroscope and the compound microscope (the latter was used to draw the arista at 200×), then inked and scanned. Photomicrographs were stacked using the software Helicon Focus Pro 6.0 (HeliconSoft Ltd.).

Morphological terminology follows that used in Roháček (2006, 2013), including terms of the male postabdomen and genitalia. The latter are largely based on the “hinge” hypothesis of the origin of the eremoneuran hypopygium, re-discovered and documented by Zatwarnicki (1996). Therefore, the next terminological changes on structures of the male genitalia are followed (synonymous terms used by other hypotheses in parentheses): epandrium (periandrium), gonostylus (surstylus, telomere).

### Abbreviations

**A1** anal vein;

**ac** acrostichal (setulae);

**ar** arista;

**C** costa;

**ce** cercus;

**Cs2, Cs3, Cs4** 2nd, 3rd, 4th costal sector;

**CuA_1_** cubitus;

**cx_1_, cx_2_, cx_3_** fore, mid, hind coxa;

**dc** dorsocentral setae;

**dm** discal medial cell;

**dm-cu** discal medial-cubital (= posterior, tp) cross-vein;

**ep** epandrium;

**f_1_, f_2_, f_3_** fore, mid, hind femur;

**fl_1_** first flagellomere;

**hu** humeral (= postpronotal) (seta);

**hum** humeral cross-vein;

**M** media;

**mspl** mesopleural (= anepisternal) (seta);

**npl** notopleural (seta);

**oc** ocellar (seta);

**ors** fronto-orbital (seta);

**p** pedicel;

**pa** postalar (seta);

**pk** preapical kink on R_1_;

**ppl** propleural (= proepisternal) (seta);

**prs** presutural (seta);

**pvt** postvertical (seta);

**R_1_** 1st branch of radius;

**R_2+3_** 2nd branch of radius;

**R_4+5_** 3rd branch of radius;

**r-m** radial-medial (= anterior, ta) cross-vein;

**S1–S10** abdominal sterna;

**sa** supraalar (seta);

**sc** scutellar (seta);

**Sc** subcosta;

**stpl** sternopleural (= katepisternal) (seta);

**T1–T10** abdominal terga;

**t_1_, t_2_, t_3_** fore, mid, hind tibia;

**vi** vibrissa;

**vte** outer vertical (seta);

**vti** inner vertical (seta).

## Systematic palaeontology

### Class Insecta Linnaeus, 1758

#### Order Diptera Linnaeus, 1758

##### Superfamily Opomyzoidea Fallén, 1820

###### Family *incertae sedis*

####### Genus Acartophthalmites

Taxon classificationAnimaliaDipteraincertae sedis

Hennig, 1965

######## Type species.

*Acartophthalmites tertiaria* Hennig, 1965; Baltic amber (mid Eocene).

####### Acartophthalmites willii sp. n.

Taxon classificationAnimaliaDipteraincertae sedis

http://zoobank.org/11E19D64-43F1-424E-9C30-59B77DDFDC04

Figures 1–2 , 3–6 , 7–10 , 11–14 , 15–16 , 17–20 , 21–22

Acartophthalmites tertiaria Hennig, 1965; Hennig 1969: 18.
Acartophthalmites electrica Hennig: Hennig 1969: 18−19, figs 19−21 [error, incorrect subsequent spelling].

######## Etymology.

The new species is named in honour of Willi Hennig (1913–1976), the founder of phylogenetic systematics and an outstanding dipterist who discovered the genus *Acartophthalmites* as well as many other fossil acalyptrates in Baltic amber.

######## Type material.

Holotype ♂, MCZ-PALE-19475. The amber piece preserving the holotype is encased in an Epoxy resin prism of 18 × 18 × 5 mm. The Epoxy prism is mounted on a glass slide with labels “Mus. Comp. Zool. 19475, No. 6545a [the latter number scratched] Haren Coll., Baltic amber”, “Fam. Acartophthalmidae. *Acartophthalmites electrica* Hennig, ♂”, and “HOLOTYPUS ♂, *Acartophthalmites willii* sp. n., R. Pérez-de la Fuente, C. Hoffeins & J. Roháček det.” (red label). Deposited in the Museum of Comparative Zoology, Harvard University, Cambridge, Massachusetts, USA.

######## Locality and age.

Baltic Sea coast, probably the Samland Peninsula (Weitschat and Wichard 2010). Ypresian to Priabonian, Eocene, 56−33.9 Ma (Weitschat and Wichard 2010).

######## Diagnosis.

Slightly smaller than both *A. tertiaria* and *A. clusioides*. Arista relatively shortly ciliate; vi relatively well-developed; two dc setae; prescutellar ac setae well developed; anterior pa very long (together with apical sc the longest thoracic seta); male f_2_ not longer than f_3_, not particularly tapered distally and ventrally with a single short row of six thicker setae; t_2_ with a long row of erect posterior setae (six or seven longer and thicker) and with three short dorsal setae (including a preapical one); wing relatively elongated and with darkening along anterior margin (in cells r_2+3_ and r_4+5_); R_1_ with a few setulae subapically; M reaching the wing margin; R_4+5_ slightly bent; dm cell elongated; apical part of CuA_1_ short, not longer than dm-cu; A_1_ relatively long, almost reaching wing margin; alula large and broad; dorsal pregenital sclerite T6+S8(?) of male short.

######## Description.

**Figures 1–2.**
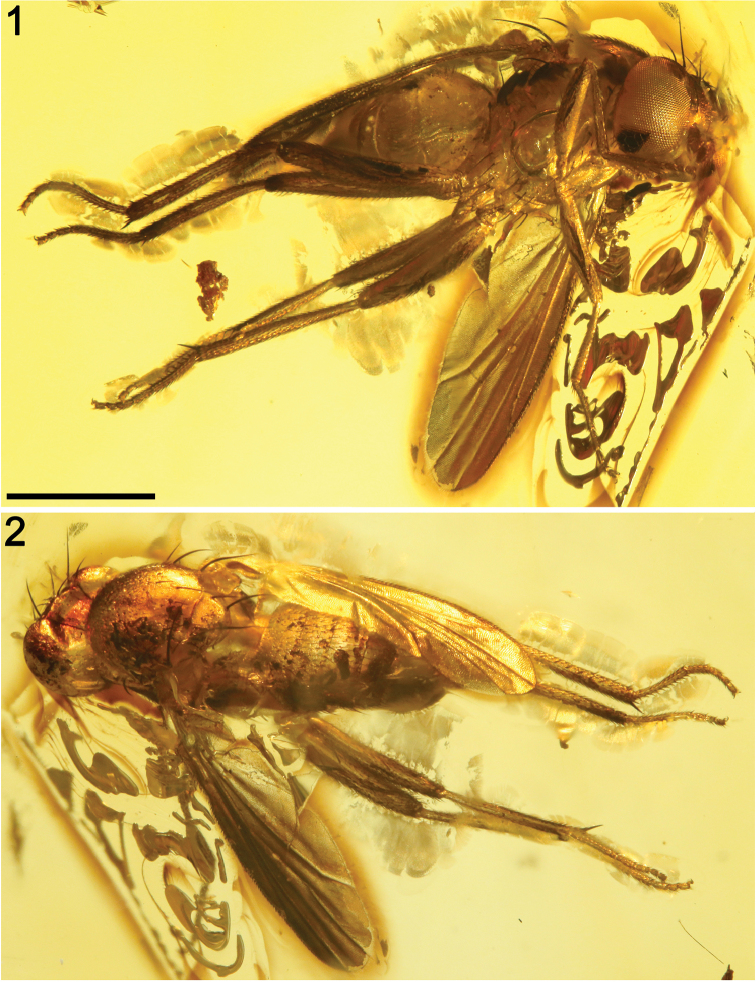
Habitus photographs of *Acartophthalmites willii* sp. n., holotype ♂ MCZ-PALE-19475 in Baltic amber (mid Eocene). **1** Ventrolateral habitus **2** Dorsolateral habitus. Scale bar 1 mm (both photographs at the same scale).

*Male* (female unknown). Total body length nearly 3.2 mm (Fig. 1); general colour probably bicoloured, dark brown to light brown and ochreous; legs light brown to ochreous.

**Figures 3–6.**
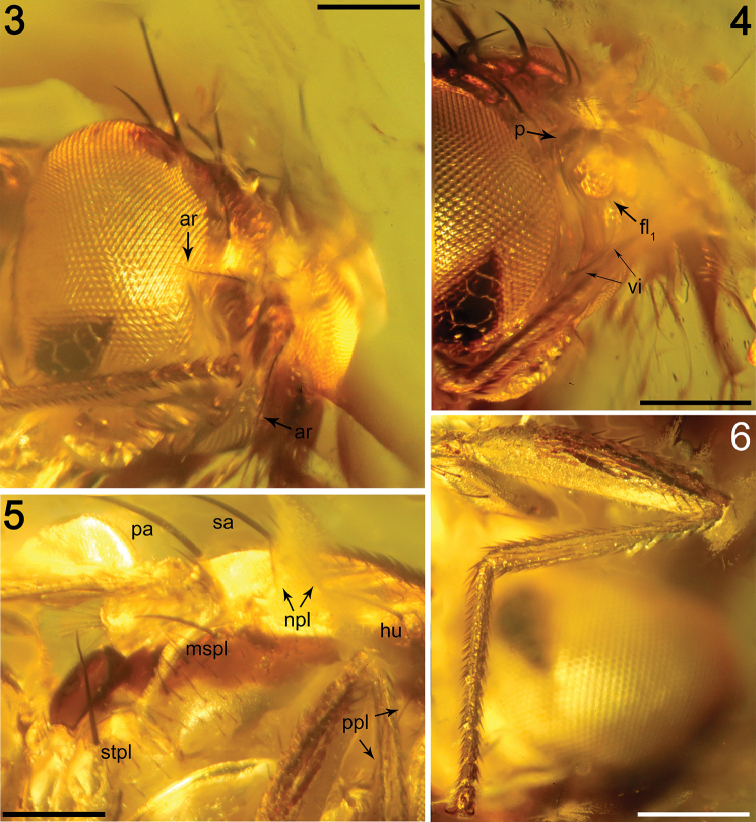
Photomicrographs of *Acartophthalmites willii* sp. n., holotype ♂ MCZ-PALE-19475 in Baltic amber (mid Eocene). **3** Head in frontolateral view **4** Head in lateral view **5** Partial thorax in lateral view **6** Right foreleg. Scale bars 250 µm.

*Head* (Figs 3–4, 15). Higher than long, 0.78 mm high, 0.66 mm long, 0.91 mm wide, dorsally somewhat wider than thorax; dorsal part of occiput concave. Head distinctly bicoloured; frons mostly light brown; occiput darkest (dark brown); face, gena and postgena ochreous. Frons moderately narrow (probably slightly wider than eye in dorsal view), slightly tapering anteriorly, largely light brown, with foremost part ochreous and only ocellar triangle dark brown. Frons rugose in texture. Orbit colouration apparently not distinct from that of frons. Frontal triangle not visible. Ocellar triangle somewhat tubercle-like, protruding among ocelli. Frontal lunule not obvious, probably small. Face (praefrons) dark ochreous, parafacialia and gena ochreous. Postgena and adjacent part of occiput large, expanded, ochreous. Cephalic chaetotaxy (Fig. 15): pvt relatively strong (longer and thicker than oc), divergent and inserted rather closely; vti very strong (longest cephalic seta), almost twice as long as vte; oc relatively weak, inserted between ocelli and directed forward; three reclinate ors becoming shorter anteriorly, the hindmost ors longest and strongest (nearly as long as vte); no microsetulae on frons medially or in front of ors; postocular setulae in a single long row surrounding posterior eye margin, none of them enlarged but there are numerous additional and erect setulae scattered on adjacent lateral parts of occiput and postgena; postgena with two or three (one distinctly longer) posteroventral setae in addition; foramen not visible; vi distinct and relatively well developed (Figs 4, 15), about twice as long as foremost peristomal setulae, curved medially; subvibrissa not developed; peristomal setulae small and sparse (four observed). Eye large, bare, strongly convex and covering most of head in profile, subovoid, its longest diameter 1.35 times as long as shortest diameter. Gena very low, its height 0.05 times as long as shortest eye diameter. Palpus ochreous, small, with ventropreapical seta the longest and a few setulae subdorsoapically. Mouthparts ochreous; labella large and fleshy, setulation not apparent. Antenna porrect and relatively small (Figs 3–4, 15), scape and pedicel light brown; pedicel externo-laterally without anterior process in the middle but with somewhat excavated anterior margin, with one stronger erect seta dorsally and two finer setae ventrally in addition to series of marginal and submarginal setulae; first flagellomere strongly laterally compressed, in profile subcircular with posterior side at least not evidently excavated, probably slightly so. Arista dorsobasal, 2.5 times as long as antenna, with elongated and whitish basal segment and darker ochreous terminal section being distinctly but relatively shortly ciliate (Figs 3, 15).

*Thorax* (Figs 5, 16). Slightly narrower than head, bicoloured, largely brown to ochreous, with some parts dark brown. Scutum ochreous, with a longitudinal medial light brown band wider proximally and distally, and two brown spots at the posterior half of the scutum which enclose the sa, pa and the two dc setae (see Fig. 16). Humeral (postpronotal) callus brown and markedly protruding; notopleural area ochreous; scutellum and subscutellum light brown; pleural part of thorax distinctly bicoloured: dorsal part of mesopleuron (anepisternum) and pteropleuron (anepimeron) dark brown; ventral part of mesopleuron and pteropleuron ochreous; metapleuron (katatergite) dark brown; metanotum (anatergite) ochreous; propleuron, sternopleuron (katepisternum), hypopleuron (meron) ochreous. Scutellum subtriangular with rounded apex, slightly convex dorsally; subscutellum well developed. Thoracic chaetotaxy (Figs 5, 16): one strong and long hu (plus a number of microsetae on humeral callus), two npl (anterior npl slightly longer than posterior npl), one long and robust sa (but much shorter than anterior pa), two pa (anterior pa very long and strong, the longest thoracic seta; posterior pa thinner and less than half the length of the anterior pa); no prs; two dc (both postsutural), anterior dc situated slightly behind level of sa, short (about half length of posterior dc), posterior dc robust but clearly shorter than apical sc or anterior pa; enlarged prescutellar ac present immediately after the level of posterior dc, subequal in length and thickness to posterior pa; scutum otherwise covered by uniform and relatively dense microsetae (around 15 dc microsetae in row in front of anterior dc); ac microsetae arranged in about eight rows in front of suture but less posteriorly, and only four rows reaching the level of posterior dc); two sc, apical sc strong and very long but shorter than anterior pa, laterobasal dc relatively robust, as long as three-fourths of the apical sc; one long ppl; mesopleuron with one distinct mspl in posterodorsal corner and numerous microsetae on most of its surface (except for anterodorsal part); sternopleuron with one long stpl and a number of scattered microsetae (anterior part of sclerite covered by a bubble); prosternum not visible.

*Legs* (Figs 6, 10–12, 18–21). Originally probably all light brown to ochreous, relatively long and slender. Fore, mid and hind legs differing in length of their segments (but not as strikingly as in *A. clusioides*). Femur, tibia and basitarsus of mid leg 1.5−1.7 times as long as those of foreleg, mid tibia only slightly longer than hind tibia (other leg segments between mid and hind legs subequal in length); cx_3_ with an acute distoventral setose process directed caudally (Fig. 10) (a similar process also is present in *A. clusioides*); f_1_ with a short row of four or five longer posteroventral to ventral setae in distal third and with about four posterodorsal setae forming a row in the middle of third of femur; f_2_ elongated, slightly thicker than f_3_ but subequal in length to the latter, not particularly tapered distally, finely densely setulose but ventrally with a single short distal row of six thicker setae; brush of ventral upright hair-like setulae in proximal half of f_2_ lacking; f_3_ without specific setae, uniformly densely finely setulose; t_1_ also uniformly finely setulose but with a few distal slightly thicker setae; t_2_ with distinctive chaetotaxy formed, besides usual short setosity, by a row of sparse erect posterior setae (six or seven longer and thicker, Fig. 20–21) starting in proximal two-fifths, three short dorsal setae (one in distal third, one at the level of distalmost posterior seta, and one preapical) and one longer and thicker ventroapical seta plus an anteroapical whirl of two or three shorter thicker setae near the latter (Fig. 19) and four smaller, short but thicker setulae also posteroapically (Fig. 20); t_3_ without dorsopreapical nor ventroapical seta, but with four longer and thicker anteroapical setae (Fig. 21), otherwise uniformly finely setulose. Tarsi simple, slender; forebasitarsus with four or five longer, thicker setae ventrobasally, setae increasing in length distally; mid and hind basitarsi long and with thicker laterally directed setulae (more apparent in mid basitarsus); claws relatively small.

**Figures 7–10.**
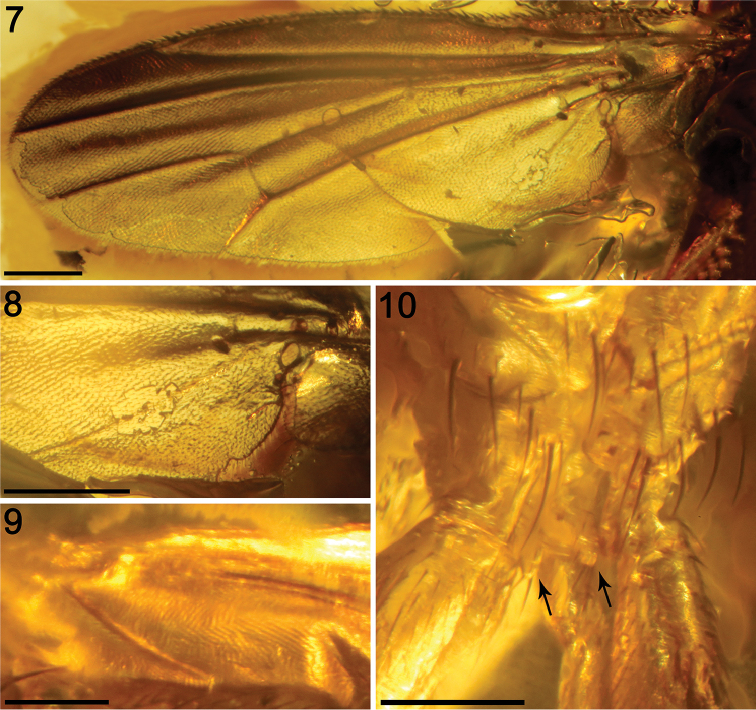
Photomicrographs of *Acartophthalmites willii* sp. n., holotype ♂ MCZ-PALE-19475 in Baltic amber (mid Eocene). **7** Left wing (slightly narrower than in reality due to the photographic angle, see Fig. 17 for an accurate wing shape) **8** Detail of the A_1_ vein from the left wing **9** Detail of the A_1_ vein from right wing **10** Sternum and basal leg segments in frontal view, showing the distoventral processes in Cx_3_ (arrows). Scale bars 250 µm.

*Wing* (Figs 7–9, 17). Elongated and narrow; veins light brown; membrane apparently darkened in the anterior third of wing, darkening more restricted to roughly the anterior half of the spaces between C and R_2+3_, and R_2+3_ and R_4+5_ (Fig. 17). C strikingly attenuated beyond R_4+5_ but this slender nebulous part reaching to M; C finely setulose but basally with a pair of longer setae and Cs_2_ (sector between apices of R_1_ and R_2+3_) with thicker (but not longer) sparse spine-like setulae in addition. No costal break. Sc fine, distally ending into C, not fused with R_1_. R_1_ short, robust and bearing four setulae subapically, preapical kink present at the level of Sc end. R_2+3_ long, very slightly sinuate, apically somewhat upcurved to C, ending distinctly farther from wing apex than M. R_4+5_ shallowly but distinctly bent posteriorly, distally subparallel with M, ending close to wing apex. Distal part of M very slightly bent and reaching wing margin (it appears as not reaching the wing margin in left wing due to preservation, but this character is well visible in the right wing). Discal (dm) cell relatively elongated; anterior cross-vein (r-m) situated in about the middle of discal cell. Distal part of CuA_1_ subequal in length to dm-cu cross-vein and reaching wing margin; A_1_ elongated, almost reaching the wing margin. Cells bm and cup closed. Anal lobe moderately developed. Alula well developed, large and broad. Wing measurements: length 2.2 mm, width 0.85 mm, Cs_3_ : Cs_4_ = 2.05, r-m\dm-cu : dm-cu = 3.24. Haltere ochreous.

*Abdomen* (Fig. 1). Piriform in dorsal view, widest at distal end of T2. All preabdominal terga rather sparsely but distinctly setose, with longest setae (some upright) at posterior and lateral margins. T1–T3 dorsally ochreous, darker laterally, T4 and T5 dark brown. T1−T2 separation not conspicuous. T1–T5 relatively narrow, only slightly bent laterally (pleural membrane rather well developed). Preabdominal sterna ochreous, sparsely and shortly setose (only S2−S3 visible, very narrow). Postabdomen (Figs 13–14, 22) with sclerites well developed, asymmetry of postabdominal sterna not clearly assessable. T6 not present as separate sclerite, either reduced (absent) or completely fused with S8 to form with it a large but not long synsclerite T6+S8(?) being dark brown, densely shortly setose. S6 and S7 not clearly discernible, but probably asymmetrical and largely situated left laterally.

**Figures 11–14.**
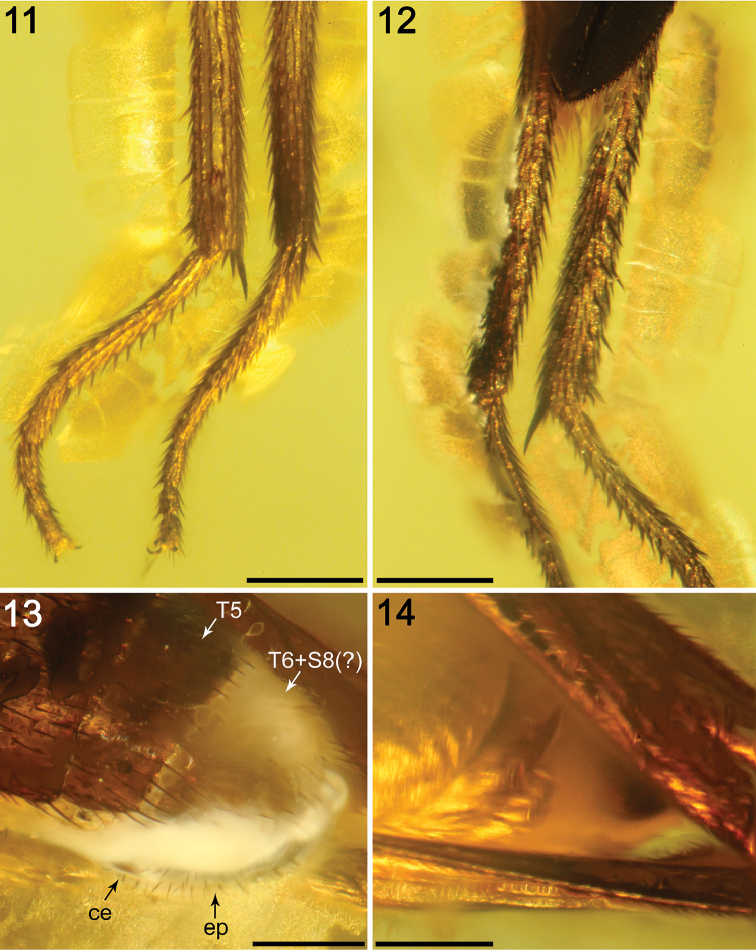
Photomicrographs of *Acartophthalmites willii* sp. n., holotype ♂ MCZ-PALE-19475 in Baltic amber (mid Eocene). **11** Right mid- (left) and hind tibiae’s distal half and tarsi (right) in anterior (i.e., lateral, external) view **12** Right mid- (right) and hind tibiae’s distal half and tarsi (left) in posterior (i.e., lateral, internal) view **13** Apex of abdomen in dorsal view **14** Apex of abdomen in ventral view. Scale bars 250 µm.

*Genitalia.* Epandrium short, width not assessable, shortly uniformly setose. Cerci barely visible and gonostyli not visible as the sample is currently prepared (but originally depicted by Hennig 1969: fig. 20; see Fig. 22). Based on his description and illustration gonostylus is simple, slender, seemingly bare and slightly bent posteriorly and cercus is elongated, shorter than gonostylus, setose, and with longer setae apically (Fig. 22). However, as the anteroventral parts of external genitalia were not visible (also to Hennig), we cannot exclude the possibility that he observed and illustrated only the posterior lobe of the left (bilobed) gonostylus. In any case, this lobe is distinctly different from that of *A. clusioides*, where it distinctly bends anteriorly (cf. Roháček 2016: fig. 17).

**Figures 15–16.**
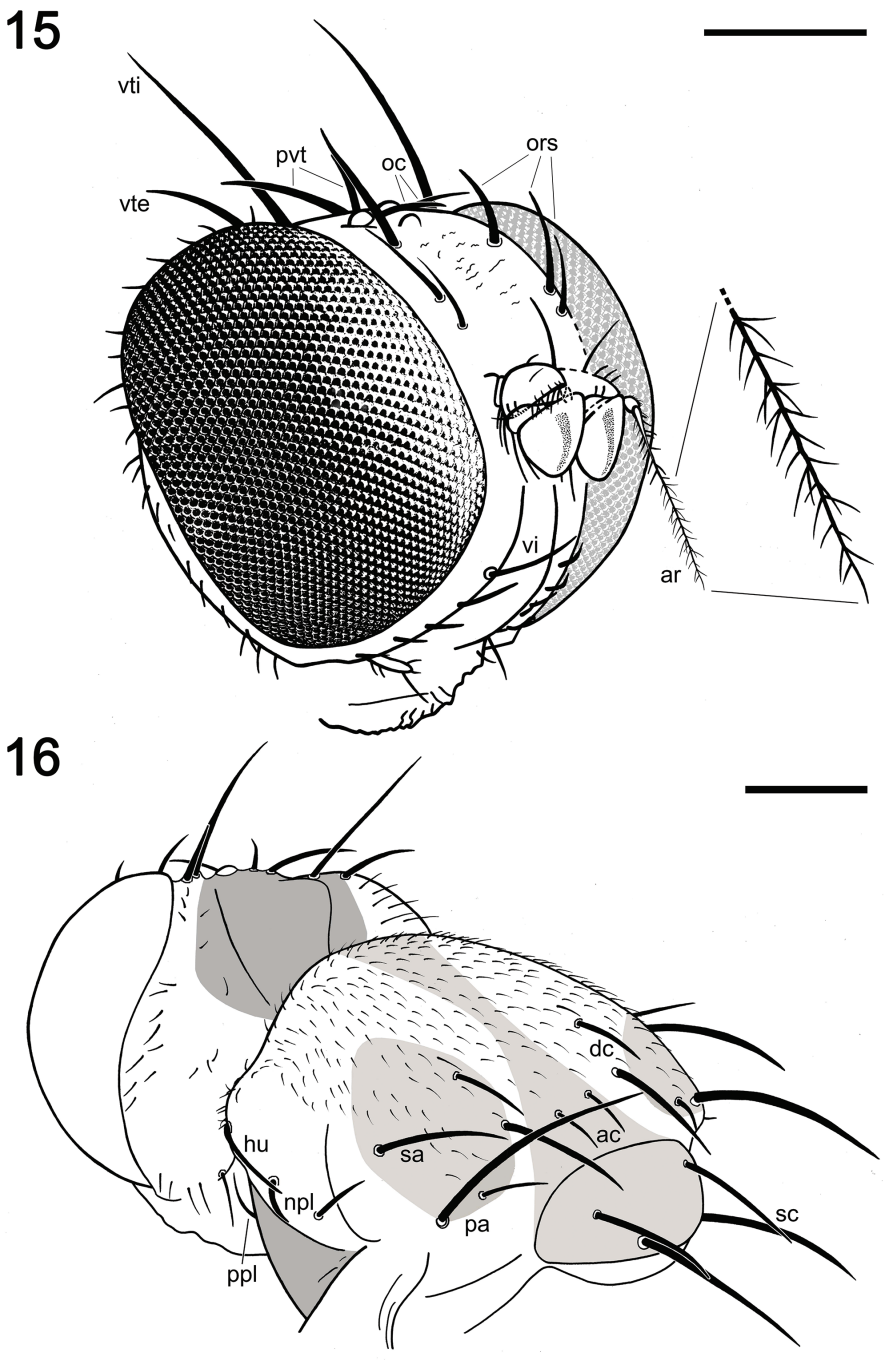
Camera lucida drawings of *Acartophthalmites willii* sp. n., holotype ♂ MCZ-PALE-19475 in Baltic amber (mid Eocene). **15** Head in frontolateral view with the cephalic chaetotaxy tagged; note the inset of the distal half of the left arista **16** Head and thorax in posterolateral view, with the thoracic chaetotaxy tagged. Scale bars 250 µm.

**Figures 17–20.**
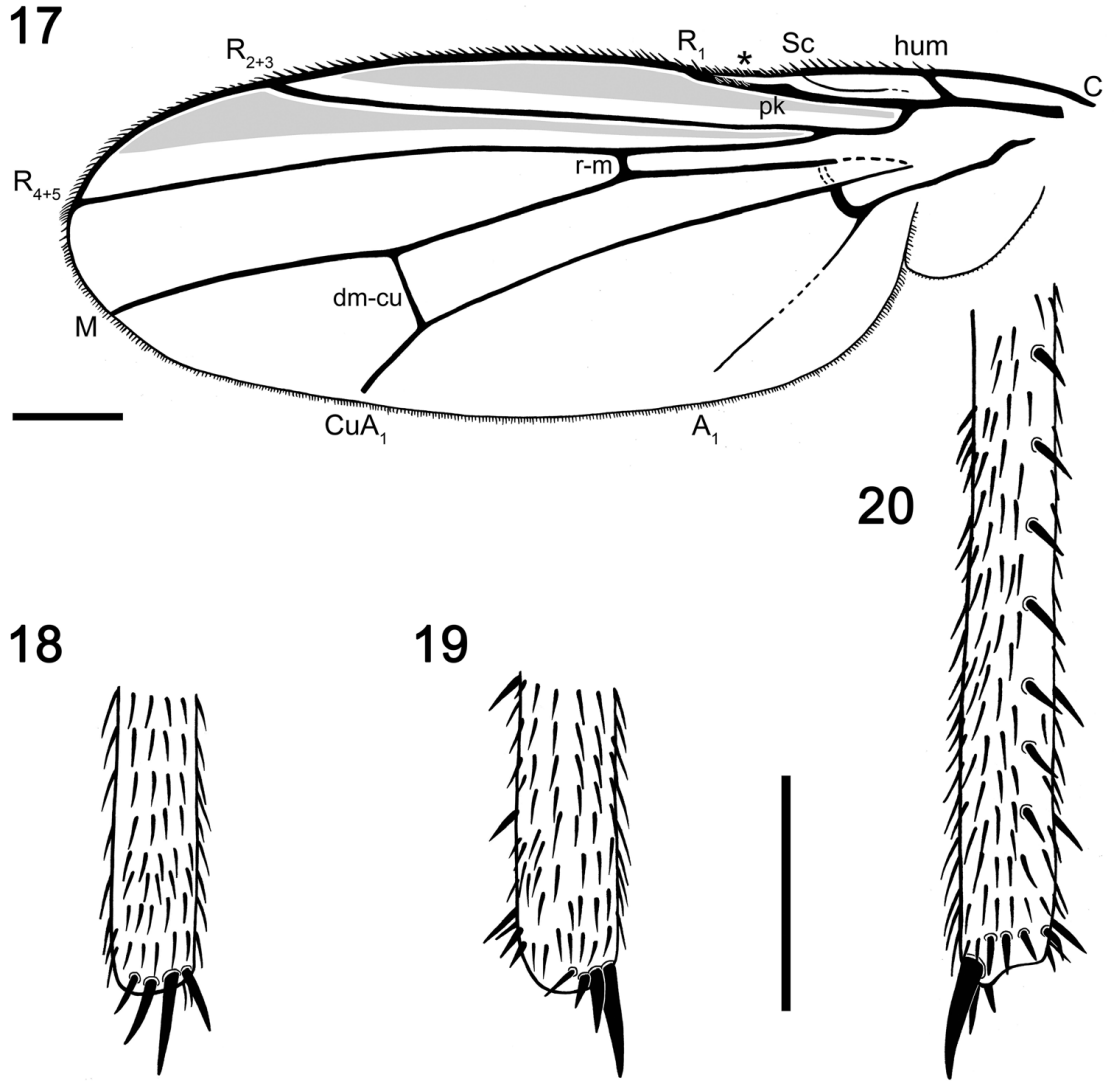
Camera lucida drawings of *Acartophthalmites willii* sp. n., holotype ♂ MCZ-PALE-19475 in Baltic amber (mid Eocene). **17** Wing with venation tagged; the asterisk marks a depressed area of the wing **18** Distal quarter of the right hind tibia in anterior (lateral, external) view **19** Distal quarter of right mid tibia in anterior (i.e., lateral, external) view **20** Distal half of the right mid tibia in posterior (i.e., lateral, internal) view. Scale bars 250 µm (**18–20** at the same scale).

**Figures 21–22.**
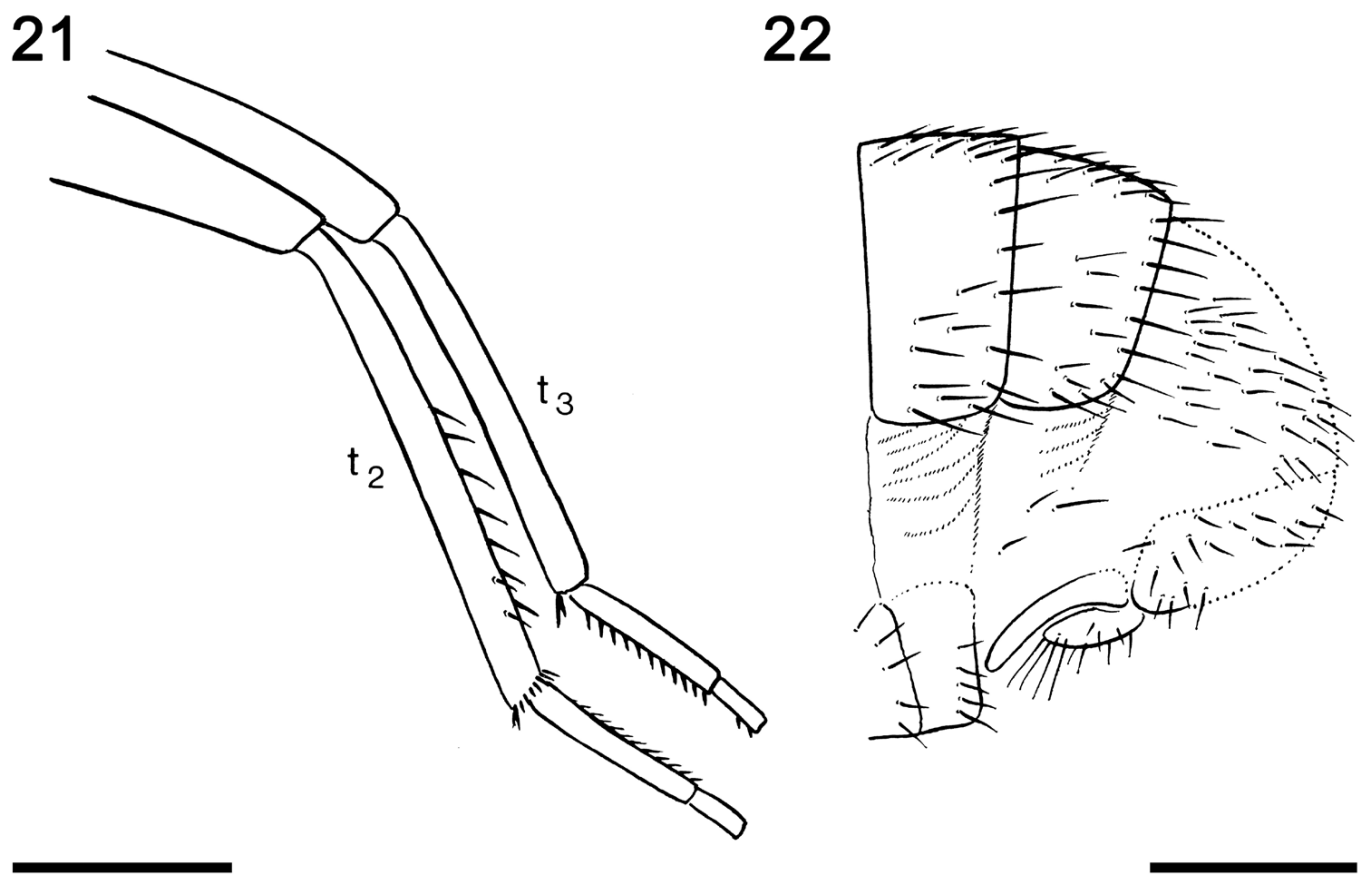
Reproductions of the original illustrations depicted by Hennig (1969, figs 20, 21) on *Acartophthalmites willii* sp. n., holotype ♂ MCZ-PALE-19475. **21** Setation of mid and hind tibiae and tarsi in anterior view (scale bar 0.5 mm) **22** Apex of abdomen in lateral view (scale bar 0.3 mm).

### Remarks

The combination “*Acartophthalmites electrica*” used by Hennig (1969) only in the caption of his illustrations (figs 19–21), and which is also written on the label from the glass slide bearing the holotype (see above), is clearly an error and needs to be considered as an “incorrect subsequent spelling” of *A. tertiaria* in the broadest sense (ICZN 1999, Article 33.3) and, consequently, an unavailable name.

*Acartophthalmites willii* sp. n. shares with *A. tertiaria* (currently known only from females, see Hennig 1965) the setulose R_1_ vein and the long A_1_ vein but differs from the latter species in the shape of the wing (distinctly more elongated), the only slightly curved R_4+5_ vein, the more elongated dm cell and the shorter terminal section of the CuA_1_ vein. Moreover, the new species’ arista has a distinctly shorter ciliation, the mesonotum bears only two dc setae and the pa setae are markedly longer (for these characters in *A. tertiaria* see Hennig 1965: figs 172, 176). In addition, the mid leg, particularly the t_2_ chaetotaxy, also seems to be very different between these two species, because Hennig (1965: fig. 189) did not describe a row of erect posterior setae nor distal dorsal setae on t_2_ in the females of *A. tertiaria*. As this species’ holotype is in very poor condition, it is unclear if these setae are weak and were overlooked by Hennig due to their short length or if they are truly absent. Based on our examination of a series of additional specimens of *Acartophthalmites*, we can now confirm that the long row of posterior setae and some dorsal ones are always present on the mid tibia in the species of this genus.

The remaining *Acartophthalmites* species known, *A. clusioides* Roháček, 2016 (based on a single male), resembles *A. willii* sp. n. in the elongated and brownish patterned wing with similar venation (including a long dm cell with a short distal section of the CuA_1_ vein). However, in *A. clusioides* the dark pattern covers a larger area of the wing (Roháček 2016: fig. 8), the R_1_ vein is completely bare, the M vein does not reach the wing margin, the A_1_ vein is short and the alula is narrower (Roháček 2016: fig. 15). Aside from body colour features, *A. clusioides* further differs from *A. willii* in having a slender body (Roháček 2016: figs 1, 2), a more elongated head (Roháček 2016: figs 5, 6, 9) with denser setation on the occiput (Roháček 2016: fig. 18), a particularly prolonged mid leg with a distally slender femur (Roháček 2016: fig. 11) and an elongated dorsal pregenital sclerite (Roháček 2016: fig. 16).

Lastly, the distoventral projection on the hind coxa described above for *A. willii* sp. n. (Fig. 10) has also been found in *A. clusioides* (while overlooked in the original description by Roháček 2016) as well as in some male *Acartophthalmites* to be described elsewhere. This structure is therefore considered a male secondary sexual character probably occurring in males of all species within the genus.

## Discussion

Hennig (1965) already pointed out some distinct differences among the specimens within the type series of *Acartophthalmites tertiaria* Hennig, 1965, and the subsequently described male that he classified in the same species (Hennig 1969). These differences and additional ones were also stressed by Roháček (2016), who suggested that at least some of these specimens belonged to one or more different species. Our reassessment of the male held at the MCZ confirmed this assumption and, therefore, it is herein described as a new species. Detailed morphological study has resulted in finding out that *A. willii* sp. n. is somewhat morphologically intermediate between *A. tertiaria* and *A. clusioides* Roháček, 2016, and, consequently, that it falls within the widened taxonomic limits of the genus *Acartophthalmites* Hennig, 1965 as defined by Roháček (2016: p. 419).

The general resemblance in body structures and wing venation to Clusiidae is also confirmed for *A. willii*. The main differences of *Acartophthalmites* when compared to Clusiidae are the relatively short vibrissa, the structure of the antennal pedicel (lacking an angulate projection at both the external and internal margins), the arista in dorsobasal position, the absence of subcostal break and the bare prosternum (see Lonsdale et al. 2010). Even though elucidating the relationships of *Acartophthalmites* is a matter of further study as additional specimens with well-visible male and female terminalia are needed, the current knowledge expanded by the present work recognises clusiid flies as the most probable relatives of this fossil genus. Consequently, the genus *Acartophthalmites* should be tentatively classified within the superfamily Opomyzoidea.

This study represents a further step towards the revision of the specimens classified within the genus *Acartophthalmites*, which is aimed at the acquisition and critical evaluation of morphological data so urgently needed for a reliable phylogenetic analysis of this lineage of acalyptrate flies.

## Supplementary Material

XML Treatment for Acartophthalmites

XML Treatment for Acartophthalmites willii
